# SFRT combined with immunotherapy for bulky hepatic metastasis from pancreatic acinar cell carcinoma: a case report

**DOI:** 10.3389/fonc.2026.1815116

**Published:** 2026-04-29

**Authors:** Da Fu, Chanjin Liang, Ling Yuan, Ying Liu, Min Zhang, Xingyuan Shi

**Affiliations:** Department of Radiotherapy, The Fifth Affiliated Hospital of Guangzhou Medical University, Guangzhou, Guangdong, China

**Keywords:** case report, hepatic metastasis, immunotherapy, PACC, SFRT

## Abstract

**Background:**

Pancreatic acinar cell carcinoma (PACC) is a rare malignancy, and clinical data on managing bulky hepatic metastases from PACC remain limited. Conventional radiotherapy is restricted by normal tissue tolerance, while stereotactic body radiation therapy (SBRT) leads to prohibitive toxicity when applied to large tumor volumes. Spatially fractionated radiation therapy (SFRT) creates a deliberate non-uniform peak-valley dose distribution, delivering ablative doses to discrete tumor vertices while sparing the surrounding hepatic parenchyma. This approach may enhance local control and antitumor immunity, supporting its use in combination with immunotherapy for large hepatic metastases.

**Case report:**

A 35-year-old female with metastatic PACC and a solitary 12-cm hepatic metastasis (clinical stage TxN3M1) experienced disease progression despite multiple surgical resections and six lines of systemic chemotherapy. The enlarging hepatic metastasis caused portal vein compression and anasarca. The patient received Lattice SFRT (40 Gy in four fractions) to the hepatic lesion, combined with the PD-1/CTLA-4 bispecific antibody cadonilimab and the multi-targeted tyrosine kinase inhibitor anlotinib. The target lesion decreased by 34% at 1 month and nearly 50% at 5 months, meeting RECIST partial response. No grade ≥3 toxicities were observed.

**Conclusion:**

This case provides clinical experience supporting the use of SFRT combined with immunotherapy for bulky refractory PACC. The combination of Lattice SFRT, dual immune checkpoint blockade, and targeted therapy appears feasible and well-tolerated in this patient. Further clinical trials are warranted to validate this combined approach.

## 1 Introduction

The liver is a predominant site of metastasis for cancers such as colorectal, breast, and lung malignancies (1). Hepatic metastases typically indicate advanced disease and are associated with complex management challenges and poor prognosis (2), especially for multiple or large lesions (≥5 cm in diameter). Current treatment options, including resection, ablation, chemotherapy, radiotherapy, and immunotherapy, each have inherent limitations in this setting. Surgical resection is often unfeasible due to vascular involvement or an insufficient future liver remnant. Ablation techniques are less effective for large tumors because of incomplete necrosis and the heat-sink effect (3). Systemic chemotherapies are hindered by poor intratumoral drug penetration and frequent resistance within the immunosuppressive tumor microenvironment. While SBRT is effective for small lesions, its application to large tumors is limited by toxicity to normal liver and adjacent organs (4).

SFRT offers a promising alternative for treating large hepatic metastases. By delivering a deliberate non-uniform peak-valley dose distribution, SFRT administers ultra-high doses (e.g., 10–20 Gy per fraction) to discrete tumor vertices while sparing most of the tumor volume and surrounding normal tissues. This unique dosing pattern induces immunogenic cell death (ICD) of tumor cells, promotes the release of tumor-associated antigens, and preserves intratumoral immune cells and vascular structures (5, 6). Thus, SFRT holds substantial potential for combination with radio-immunotherapy regimens to synergistically enhance antitumor efficacy.

Lattice, a form of SFRT, delivers high-dose spheres within the tumor while maintaining low doses to adjacent tissue. This unique dose distribution may achieve both local debulking and immune modulation, thereby triggering both local response and abscopal effects. High-dose peaks induce tumor cell death, while low-dose valleys modulate the TME and enhance anti-tumor immunity (7, 8).

We present a heavily pretreated patient with bulky PACC hepatic metastasis and critical clinical compromise who underwent Lattice SFRT plus IMRT combined with anlotinib and cadonilimab.

## 2 Case presentation

A 35-year-old female presented with acute abdominal pain in September 2020, and cross-sectional imaging revealed a large abdominal mass measuring 125 × 124 × 164 mm. She underwent primary tumor resection, and postoperative pathology reviewed at the Department of Pathology, Shanghai Jiao Tong University, confirmed PACC, a rare entity accounting for 0.2%–4.3% of pancreatic cancers (9). Due to the high risk of recurrence, adjuvant chemotherapy with albumin-bound paclitaxel plus gemcitabine was initiated. However, multiple intra-abdominal metastases developed within months, and second-line therapy with bevacizumab and tislelizumab also failed to control disease progression.

A treatment shift to anlotinib combined with tislelizumab marked a turning point. Follow-up imaging in April 2021 showed significant tumor regression, and concurrent genetic testing identified a germline PALB2 mutation, which guided subsequent targeted therapy. The patient then received intensive treatment with oxaliplatin, tegafur, anlotinib, and tislelizumab, followed by maintenance therapy. By October 2021, the hepatic metastatic lesions had nearly resolved, indicating a deep and sustained clinical remission.

Nevertheless, the disease progressed to a multi-refractory phase in late 2022. Despite aggressive interventions, including olaparib targeting the PALB2 mutation, debulking surgery for pelvic metastases, and multiple cycles of hepatic arterial infusion chemotherapy, only a transient remission was achieved, and disease progression (PD) was observed in both the liver and pelvic regions. In January 2025, the patient was referred to our center for further management. Upper abdominal MRI confirmed PD, demonstrating enlargement of most hepatic metastases except for mild regression of the segment VII lesion, accompanied by progression of a left anterior pelvic lesion. Hepatic arterial chemoembolization with oxaliplatin and tislelizumab was performed on January 17, 2025. The patient discontinued all anticancer treatments from February to April 2025.

**Figure 1:**
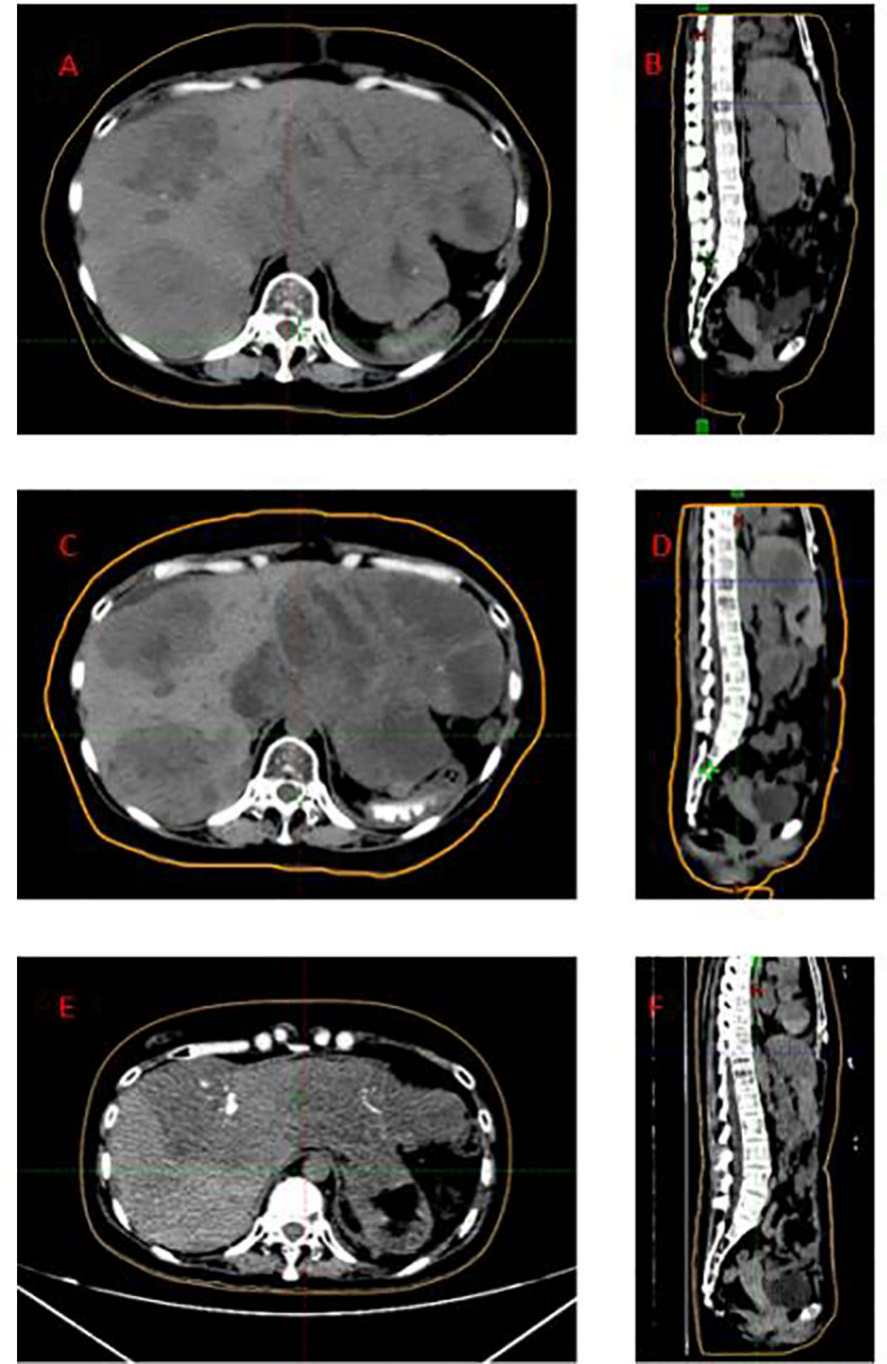
Sequential CT images demonstrating tumor response over time. **(A, B)** In April 17, 2025, the left lobe measuring up to 137 × 127 mm. **(C, D)** In May 20, 2025, the left hepatic lobe tumor to 121 × 97 mm. **(E, F)** In September 1, 2025, the left hepatic lobe mass to 110 × 87 mm, with slight shrinkage of other lesions and regression of hilar lymph nodes. The patient was in good general condition, without abdominal tenderness or lower limb edema.

On April 17, 2025, the patient developed severe abdominal and bilateral lower extremity edema. Imaging on April 21 demonstrated hepatomegaly, with the left lobe measuring up to 137 × 127 mm (**Figures 1A, B**), a 70 × 70 × 44 mm mass at the hepatic portal, a 22 × 19 mm retroperitoneal lymph node, and enlarged pelvic metastases. Vascular studies excluded thrombosis. Severe abdominal and bilateral lower extremity edema was attributed to impaired venous outflow secondary to progressive multiple hepatic, hepatic hilar and retroperitoneal lymph node metastases.

A multidisciplinary team recommended a combined regimen of radiotherapy, targeted therapy, and dual immune checkpoint blockade, and anlotinib was reinitiated. The radiotherapy plan included three components: Lattice SFRT to the left hepatic lobe metastasis (40 Gy/4 fractions) to alleviate portal hypertension, IMRT to the hepatic hilar metastasis (30 Gy/10 fractions), and IMRT to the left abdominal wall metastasis (30 Gy/10 fractions) with a simultaneous integrated boost(SIB) to 40 Gy for three embedded high-dose spheres. Immunotherapy with cadonilimab (250 mg) was initiated on April 29, followed by maintenance with cadonilimab plus anlotinib starting on May 3. The combined Lattice SFRT and hepatic hilar IMRT plan are provided in **Supplementary Figure 1**, and the IMRT plan for the left abdominal wall metastasis is provided in **Supplementary Figure 2**.

**Figure 2:**
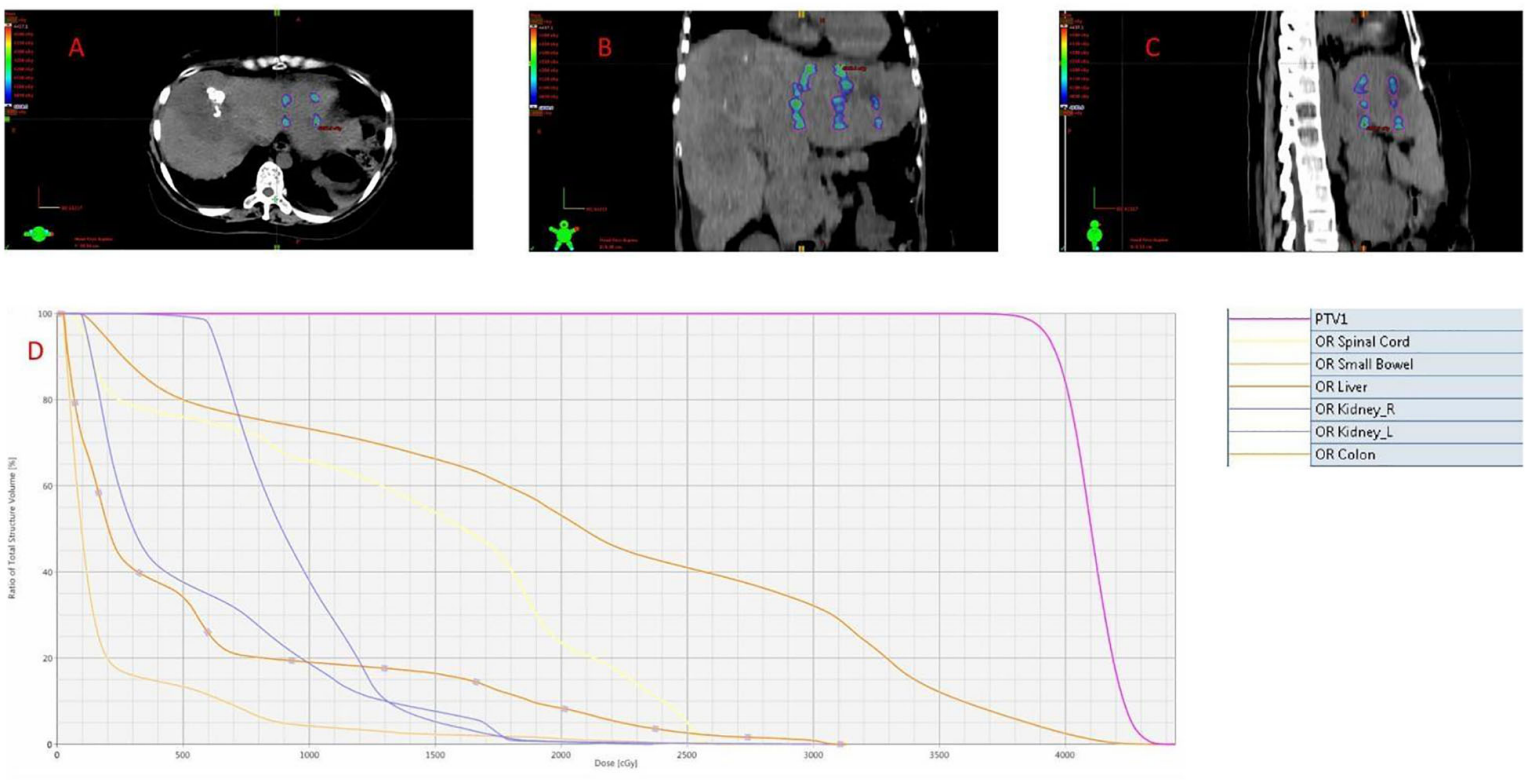
Treatment planning for Lattice SFRT of the left hepatic lobe bulky metastasis. **(A–C)** Axial, coronal, and sagittal views showing the disposition of 24 high-dose spherical vertices (1 cm diameter) within the target lesion. **(D)** Composite dose volume histogram (DVH) of the combined Lattice SFRT and hepatic hilar IMRT plan, reflecting the total dose distribution to the target volumes and adjacent organs at risk (OARs), including the spinal cord, small bowel, normal liver parenchyma, bilateral kidneys, and colon.

For the bulky left hepatic lobe metastasis, no conventional GTV or PTV was delineated due to the lesion’s close proximity to the stomach and risk of high-dose exposure to adjacent organs. Instead, 24 spherical high-dose vertices (1 cm diameter) were directly delineated and prescribed to achieve an intentionally heterogeneous peak–valley dose distribution (**Figure 2**). The spheres were arranged in a craniocaudal sequence of 4, 6, 7, and 7 vertices to cover the lesion (137 × 127 mm in cross-section, 83 mm craniocaudal length). A nominal 3 cm center-to-center spacing was adopted, consistent with the clinically validated range of 2–6 cm reported in the systematic review by Iori et al (6). The spacing was increased to 4.5 cm near critical structures. The 1 cm sphere diameter was selected based on the standard range of 0.8–1.5 cm in clinical Lattice SFRT practice. Each sphere was prescribed 40 Gy in 4 fractions, and treatment was delivered every other day under CBCT guidance. The plan achieved a peak dose of 40–44 Gy within each spherical vertex, with a valley dose of approximately 25 Gy in the intervening tumor regions.

Treatment was initiated on April 25, 2025. On May 2, gram-negative bacilli were isolated from blood culture, prompting temporary suspension of radiotherapy and initiation of empirical antibiotic therapy with ticarcillin-clavulanate. The patient became afebrile by May 5, and follow-up CBCT on May 8 demonstrated early shrinkage of hepatic and pelvic metastases with marked resolution of anasarca. Due to anatomical changes from tumor regression, the remaining five fractions of IMRT for the hepatic hilar and left abdominal wall targets were replanned with reduced fields, and the SIB to the abdominal wall lesion was omitted. Radiotherapy was resumed, and the patient reported only mild, occasional fatigue as a treatment-related symptom. Sphingomonas was isolated from a repeat blood culture on May 9, and antibiotic therapy was adjusted to levofloxacin based on susceptibility testing. The entire radiotherapy course was completed on May 14, 2025.

All treatments were delivered using a Varian Trilogy linear accelerator. Planning was performed with Eclipse TPS (v13.6), and rigorous quality assurance (QA) was conducted in accordance with institutional SBRT/IMRT protocols. All treatment plans were independently reviewed and approved by a senior radiation oncologist and a medical physicist prior to delivery.

The patient tolerated the entire treatment regimen well, with no significant radiation-related adverse events. Follow-up imaging on May 20 showed a reduction in the left hepatic lobe tumor to 121 × 97 mm (**Figures 1C, D**), with mild regression of other metastatic nodules. Additional cycles of cadonilimab plus anlotinib were administered on May 30 and June 27. A follow-up CT scan on September 1 demonstrated further reduction of the left hepatic lobe mass to 110 × 87 mm (**Figure 1E**), with mild shrinkage of other metastatic lesions and regression of hilar lymph nodes. At the last follow-up, the patient was in a good general condition with complete resolution of abdominal tenderness and lower limb edema (**Figure 1F**).

The neutrophil-to-lymphocyte ratio (NLR) was serially monitored during treatment. At baseline (April 17, 2025), the NLR was 21.74, indicating severe immunosuppression and systemic inflammation. After initiation of Lattice SFRT, cadonilimab, and anlotinib, the NLR declined rapidly: 20.29 (April 29), 38.00 (May 2, during transient infection), 3.57 (May 9), 2.09 (May 29), 2.00 (June 26), and finally 1.15 (August 31). The sustained and marked decrease in NLR indicated progressive activation of the antitumor immune response and was consistent with the observed radiological tumor regression. Neutrophil count, lymphocyte count, and NLR are provided in **Supplementary Table 1**.

**Figure 3:**
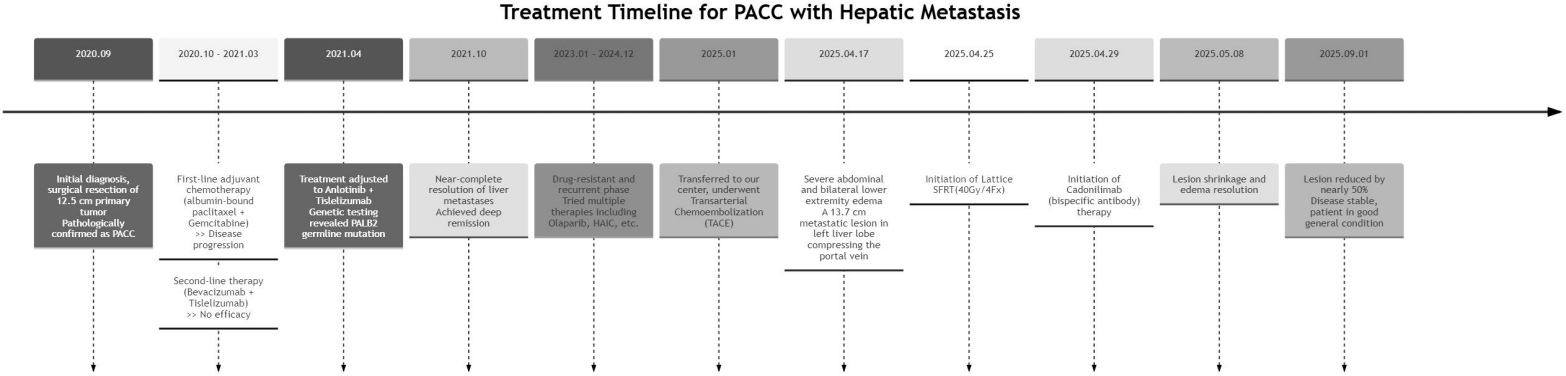
Treatment timeline of the patient with PACC.

The key time points of the patient’s treatment course are summarized in **Figure 3**.

## 3 Discussion

PACC is an exceptionally rare entity, accounting for only 0.2%–4.3% of all pancreatic malignancies. Although patients with PACC generally exhibit more favorable survival (median 18 to 30 months; 17 months for metastatic disease treated with chemotherapy) than those with pancreatic ductal adenocarcinoma (median 6 months), distant metastasis remains frequent. In fact, up to half of patients present with metastatic disease at diagnosis, and recurrence rates as high as 72% have been documented (10). Due to its rarity, there is no standardized consensus on the management of bulky, multi-refractory hepatic metastases (11). The present case illustrates the potential utility of a novel synergistic regimen combining SFRT with dual immune checkpoint inhibition and targeted therapy in a patient with refractory disease who had exhausted all standard therapeutic options.

The management of a 12-cm hepatic metastasis presents a significant technical challenge for traditional radiotherapy. Conventional SBRT is generally contraindicated in such cases due to the high risk of radiation-induced liver disease (RILD) (12). Lattice SFRT overcomes these limitations by delivering ultra-high doses to discrete 1-cm spherical vertices within the tumor volume while sparing the surrounding normal liver parenchyma. This approach generates a characteristic peak-valley dose distribution that achieves effective local tumor debulking while minimizing toxicity to the normal liver, thus avoiding RILD. In our patient, this regimen reduced the left hepatic lobe lesion from 137 × 127 × 83 mm to 110 × 87 × 64 mm (an approximate 60% volume reduction) and successfully reversed disease progression with rapid relief of clinical symptoms.

A multicenter trial (13) of palliative Lattice therapy combined with IMRT/VMAT in stage IV patients reported excellent symptom control: immediate symptom relief was observed in 3 of 30 patients, and 27 patients experienced significant improvement within 8 days post-treatment. The complete response rate was 23%, with a 1-year OS rate of 86.4%. Acute treatment-related toxicity was minimal, with only one grade 2 adverse event and no grade ≥3 toxicities recorded. Our patient achieved rapid resolution of portal hypertension-related anasarca and a 60% tumor volume reduction within five months, consistent with the preliminary positive findings from the LATTICE_01 study and further validating the efficacy and safety of Lattice SFRT for bulky metastatic lesions.

Combining SFRT with immunotherapy is an emerging research focus. High-dose radiation to the Lattice vertices triggers immunogenic cell death, facilitating the release of tumor antigens and acting as an *in situ* vaccine. Unlike whole-tumor irradiation, which may deplete local lymphocyte populations, SFRT preserves the peritumoral immune microenvironment and vasculature, allowing for the recruitment of activated T-cells. Bergeron et al. (14) suggested that non-uniform radiation may promote a spatially diversified anti-tumor immune response, potentially enhancing the therapeutic index when whole-tumor irradiation is not feasible. Jagodinsky et al. (15) demonstrated in mice that heterogeneous high-dose-rate brachytherapy synergized with dual immune checkpoint blockade (anti-PD-L1 and anti-CTLA-4), inducing spatial immune heterogeneity and enhancing CD8+ T-cell function. The peak-to-valley ratio in SFRT may optimally combine high and low doses to damage tumor while activating immunity, maximizing abscopal and bystander effects.

The choice of combination regimen was driven by the patient’s critical clinical condition, including portal compression, severe abdominal distension, and lower extremity edema, which required both rapid symptomatic relief and durable disease control. Anlotinib, a multi-target tyrosine kinase inhibitor (TKI), has been shown to improve disease control and survival when combined with immunotherapy and chemotherapy in pancreatic cancer (16). Cadonilimab, a PD-1/CTLA-4 bispecific antibody, has demonstrated promising activity in combination with SBRT for advanced refractory solid tumors, including PD-1-resistant disease, with an objective response rate (ORR) of 23.8% (17). Given the urgent need for tumor debulking and immune activation in this bulky, multi-refractory metastasis, we combined Lattice SFRT and conventional IMRT with anlotinib and cadonilimab. The patient achieved rapid and marked relief of abdominal distension and edema within two weeks after radiotherapy, indicating the immediate contribution of local radiotherapy to symptom control.

We also observed systematic immune activation during treatment, as reflected by serial changes in the NLR. NLR is a well-established marker of systemic inflammation, immune function, and prognosis in cancer, with lower values generally associated with better treatment response (18, 19). In this case, the NLR decreased persistently from 21.74 at baseline to 1.14 at three months after radiotherapy, accompanied by continuous shrinkage of hepatic and abdominal wall metastases on imaging. These findings suggest sustained anti-tumor immune activation and support the effectiveness of the maintenance strategy combining targeted therapy and dual immune checkpoint inhibition.

This case has several limitations. First, given its single-institution, single-patient design, the findings are hypothesis-generating rather than definitive. Second, as this represented an exploratory salvage strategy delivered in an urgent clinical setting, we could not delineate the relative contributions of lattice SFRT, conventional IMRT, cadonilimab, and anlotinib to the observed clinical and radiologic response. No formal analysis was performed to quantify the individual contribution of each modality or to characterize the immunological mechanisms. Third, although combination chemotherapy is conventionally preferred to improve response rates in large tumors, the patient declined chemotherapy, and a chemotherapy-free regimen incorporating anlotinib was therefore administered. Fourth, clinical evidence supporting Lattice SFRT combined with immunotherapy remains limited in pancreatic cancer, particularly for rare subtypes such as PACC. Fifth, while adverse events were monitored and no treatment-related adverse events of grade ≥3 were observed, systematic collection and standardized grading of all adverse events were not performed, limiting a comprehensive toxicity analysis.

Despite these limitations, our observations support the continued evolution and broader clinical exploration of SFRT. In addition to established LATTICE and GRID platforms, emerging techniques such as SBRT-PATHY have been developed to selectively target hypoxic tumor regions (20), with the aim of amplifying bystander and abscopal effects while maintaining the peritumoral immune microenvironment. MRI-guided adaptive SFRT has also been increasingly investigated to enable more accurate, hypoxia-informed vertex placement (21). Our experience in this rare and challenging clinical case aligns with these evolving concepts and provides further support for the clinical implementation and validation of optimized SFRT-based combination strategies.

## 4 Conclusion

In conclusion, this case offers important clinical experience suggesting that SFRT plus dual-checkpoint immunotherapy may provide meaningful local control and systemic relief in patients with bulky, refractory PACC. While firm conclusions on its safety and efficacy cannot be drawn, this approach holds certain reference value for clinical practice. Additional prospective studies are needed to verify the effectiveness of SFRT combined with immunotherapy in rare advanced malignancies.

## Data Availability

The raw data supporting the conclusions of this article will be made available by the authors, without undue reservation.

## References

[1] Aitken KL Hawkins MA . Stereotactic body radiotherapy for liver metastases. Clin Oncol (R Coll Radiol). (2015) 27(6):307–315. doi: 10.1016/j.clon.2015.01.032. PMID: 25682933

[2] Elias D Viganò L Orsi F Scorsetti M Comito T Lerut J . New perspectives in the treatment of colorectal metastases. Liver Cancer. (2016) 6(1):90–98. doi: 10.1159/000449492. PMID: 27995093 PMC5159732

[3] Ozen M Raissi D . Current perspectives on microwave ablation of liver lesions in difficult locations. J Clin Imaging Sci. (2022) 12:61. doi: 10.25259/JCIS_126_2022. PMID: 36601606 PMC9805601

[4] Guha C Kavanagh BD . Hepatic radiation toxicity: avoidance and amelioration. Semin Radiat Oncol. (2011) 21(4):256–263. doi: 10.1016/j.semradonc.2011.05.003. PMID: 21939854 PMC3434677

[5] McMillan MT Khan AJ Powell SN Humm J Deasy JO Haimovitz-Friedman A . Spatially fractionated radiotherapy in the era of immunotherapy. Semin Radiat Oncol. (2024) 34(3):276–283. doi: 10.1016/j.semradonc.2024.04.002. PMID: 38880536 PMC12013776

[6] Iori F Cappelli A D'Angelo E Cozzi S Ghersi SF De Felice F . Lattice radiation therapy in clinical practice: a systematic review. Clin Transl Radiat Oncol. (2022) 39:100569. doi: 10.1016/j.ctro.2022.100569. PMID: 36590825 PMC9800252

[7] Cho YB Yoon N Suh JH Scott JG . Radio-immune response modelling for spatially fractionated radiotherapy. Phys Med Biol. (2023) 68(16):165010. doi: 10.1088/1361-6560/ace819. PMID: 37459862 PMC10409909

[8] Amendola BE Perez NC Mayr NA Wu X Amendola M . Spatially fractionated radiation therapy using lattice radiation in far-advanced bulky cervical cancer: a clinical and molecular imaging and outcome study. Radiat Res. (2020) 194(6):724–736. doi: 10.1667/RADE-20-00038.1. PMID: 32853384

[9] Ohara Y Oda T Enomoto T Hisakura K Akashi Y Ogawa K . Surgical resection of hepatic and rectal metastases of pancreatic acinar cell carcinoma (PACC): a case report. World J Surg Oncol. (2018) 16(1):158. doi: 10.1186/s12957-018-1457-8. PMID: 30075727 PMC6091145

[10] Nasser F Motta Leal Filho JM Affonso BB Galastri FL Cavalcante RN Martins DLN . Liver metastases in pancreatic acinar cell carcinoma treated with selective internal radiation therapy with Y-90 resin microspheres. Case Rep Hepatol. (2017) 2017:1847428. doi: 10.1155/2017/1847428. PMID: 29158927 PMC5660797

[11] Sugata S Yamaguchi A Kamada H Semba S Kato N Teraoka Y . Metastatic pancreatic acinar cell carcinoma with BRCA2 gene alternation resected after modified FORFIRINOX therapy: a case report and literature review. J Gastrointest Oncol. (2025) 16(2):726–737. doi: 10.21037/jgo-24-845. PMID: 40386586 PMC12078823

[12] De La Pinta Alonso C . Radiation-induced liver disease in the era of SBRT: a review. Expert Rev Gastroenterol Hepatol. (2020) 14(12):1195–1201. doi: 10.1080/17474124.2020.1814744. PMID: 32886888

[13] Ferini G Parisi S Lillo S Viola A Minutoli F Critelli P . Impressive results after “Metabolism-guided” lattice irradiation in patients submitted to palliative radiation therapy: preliminary results of LATTICE_01 multicenter study. Cancers (Basel). (2022) 14(16):3909. doi: 10.3390/cancers14163909. PMID: 36010902 PMC9406022

[14] Bergeron P Milliat F Deutsch E Mondini M . Heterogeneous intratumor irradiation: a new partner for immunotherapy. Oncoimmunology. (2024) 13(1):2434280. doi: 10.1080/2162402X.2024.2434280. PMID: 39589158 PMC11601051

[15] Jagodinsky JC Vera JM Jin WJ Shea AG Clark PA Sriramaneni RN . Intratumoral radiation dose heterogeneity augments antitumor immunity in mice and primes responses to checkpoint blockade. Sci Transl Med. (2024) 16(765):eadk0642. doi: 10.1126/scitranslmed.adk0642. PMID: 39292804 PMC11522033

[16] Sha H Tong F Ni J Sun Y Zhu Y Qi L . First-line penpulimab (an anti-PD1 antibody) and anlotinib (an angiogenesis inhibitor) with nab-paclitaxel/gemcitabine (PAAG) in metastatic pancreatic cancer: a prospective, multicentre, biomolecular exploratory, phase II trial. Signal Transduct Target Ther. (2024) 9(1):143. doi: 10.1038/s41392-024-01857-6. PMID: 38844468 PMC11156675

[17] Xiao Y Wang Y Li J Cheng C Song C Wang X . Stereotactic body radiotherapy plus cadonilimab (PD-1/CTLA-4 bispecific antibody) as third-line or beyond therapy for refractory solid tumors: a phase 1b study. Cancer Commun (Lond). (2025) 45(10):1235–1246. doi: 10.1002/cac2.70051. PMID: 40693391 PMC12531412

[18] Xiong S Dong L Cheng L . Neutrophils in cancer carcinogenesis and metastasis. J Hematol Oncol. (2021) 14(1):173. doi: 10.1186/s13045-021-01187-y. PMID: 34674757 PMC8529570

[19] Heshmat-Ghahdarijani K Sarmadi V Heidari A Falahati Marvasti A Neshat S Raeisi S . The neutrophil-to-lymphocyte ratio as a new prognostic factor in cancers: a narrative review. Front Oncol. (2023) 13:1228076. doi: 10.3389/fonc.2023.1228076. PMID: 37860198 PMC10583548

[20] Tubin S Khan MK Salerno G Mourad WF Yan W Jeremic B . Mono-institutional phase 2 study of innovative stereotactic body radiotherapy targeting partial tumor hypoxic (SBRT-PATHY) clonogenic cells in unresectable bulky non-small cell lung cancer: profound non-targeted effects by sparing peri-tumoral immune microenvironment. Radiat Oncol. (2019) 14(1):212. doi: 10.1186/s13014-019-1410-1. PMID: 31771654 PMC6878646

[21] Dincer N Ugurluer G Korkmaz L Serkizyan A Atalar B Gungor G . Magnetic resonance imaging-guided online adaptive lattice stereotactic body radiotherapy in voluminous liver metastasis: two case reports. Cureus. (2022) 14(4):e23980. doi: 10.7759/cureus.23980. PMID: 35541303 PMC9084247

