# Supplementary material for: SFRT combined with immunotherapy for bulky hepatic metastasis from pancreatic acinar cell carcinoma: a case report
Source: Front Oncol. 2026 Apr 29;16:1815116. doi: 10.3389/fonc.2026.1815116 (PMC13167440; doi:10.3389/fonc.2026.1815116)
Supplement: Supplementary file 1 [file DataSheet1.docx]

Supplementary Material

# Supplementary Figures and Tables

## Supplementary Figures


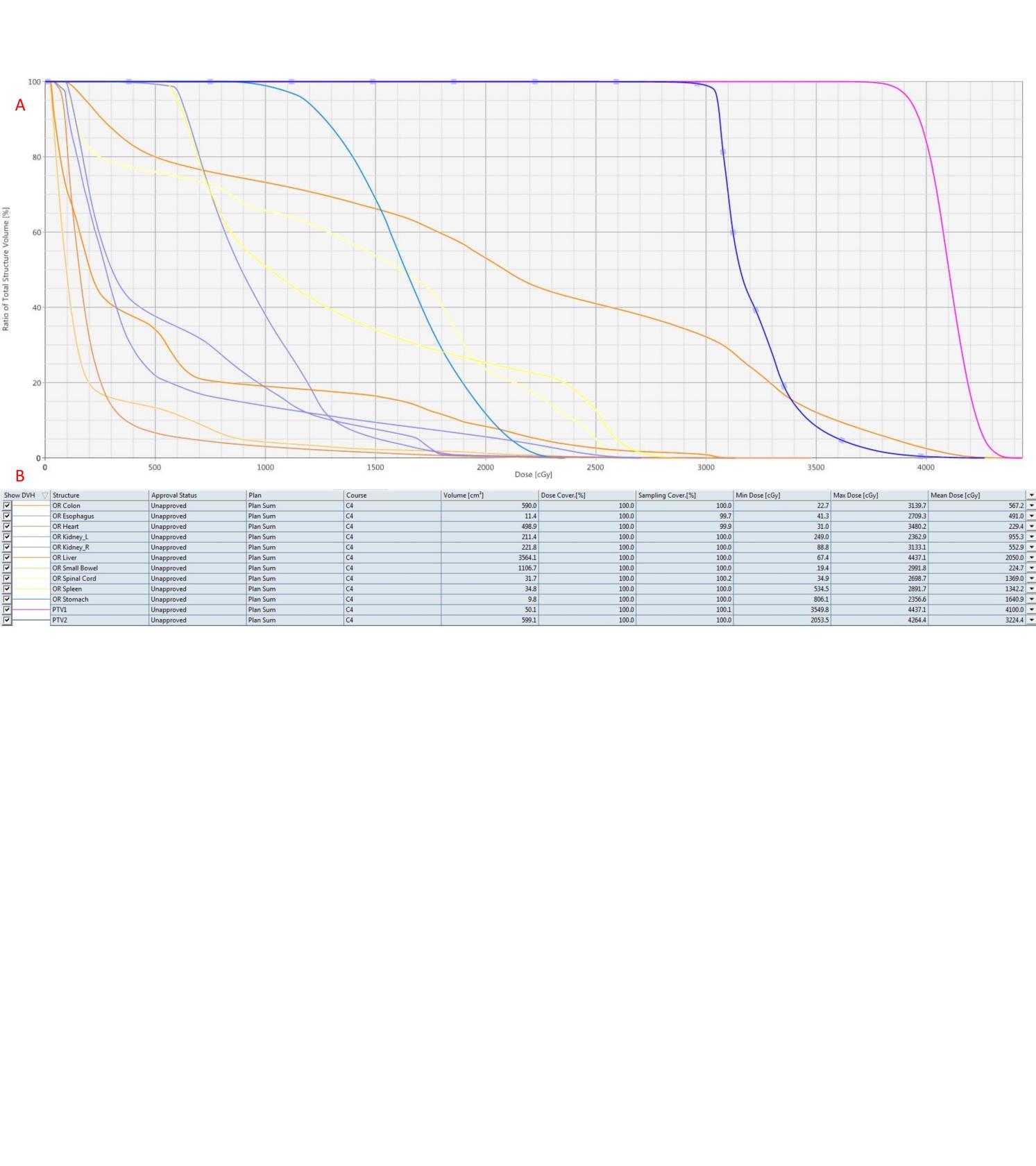


**Supplementary Figure 1.** (A) Complete DVH of the integrated Lattice SFRT and IMRT plan, including PTV1 (24 spherical high-dose vertices,Lattice SFRT high-dose spherical vertices) and PTV2 (hepatic hilar IMRT target). (B) Summary of dosimetric parameters, including the minimum, maximum, and mean doses for the PTV and organs at risk.

**
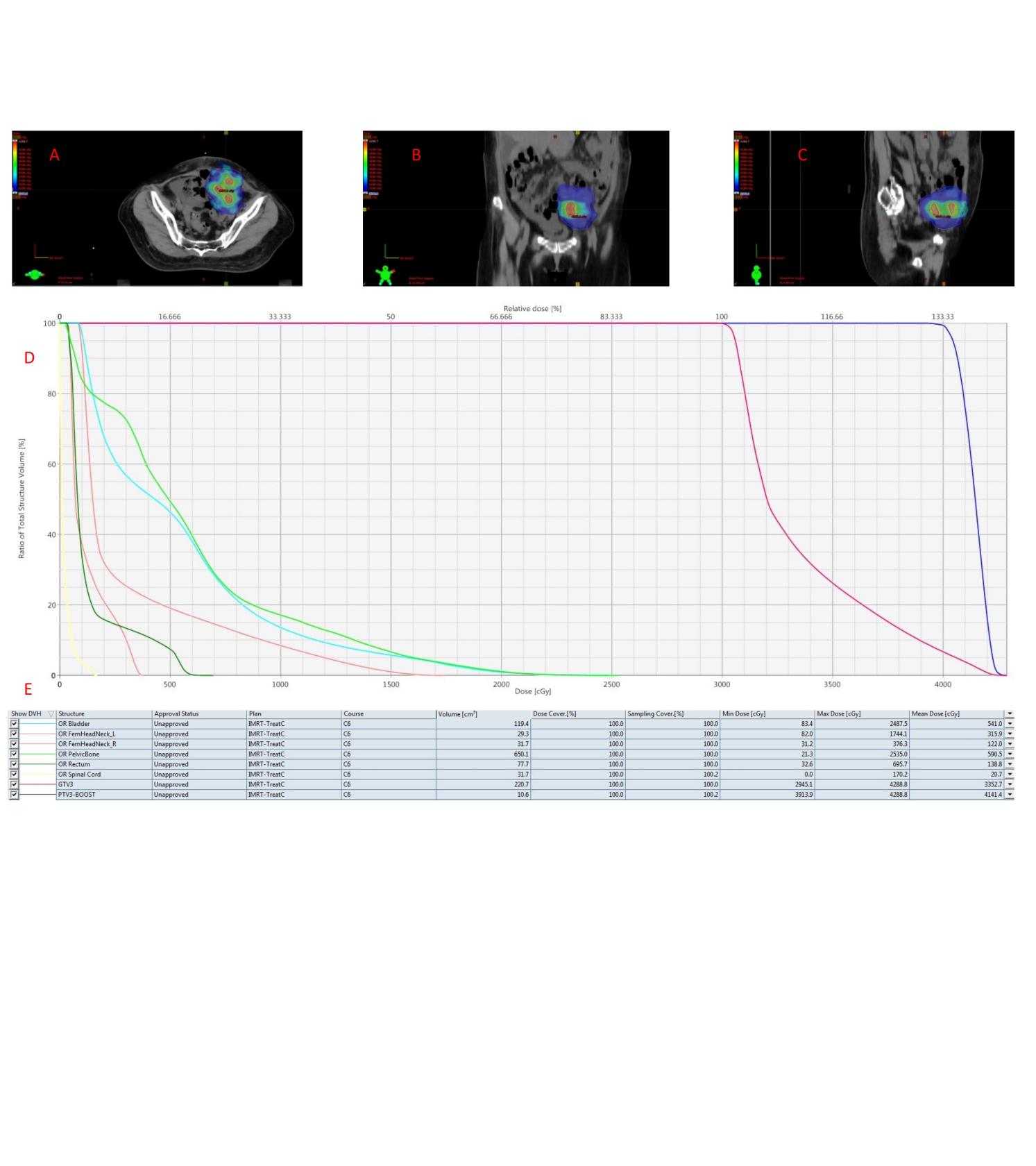
**

**Supplementary Figure 2.** IMRT plan for the left abdominal wall metastasis.

(A–C) Axial, coronal, and sagittal views showing the target volume definition (GTV, PTV BOOST) for the left abdominal wall lesion. (D) DVH of the IMRT plan, including the PTV and adjacent organs at risk. (E) Summary of dosimetric parameters, including the minimum, maximum, and mean doses for the PTV and organs at risk. The lesion was treated with 30 Gy in 10 fractions, with a simultaneous integrated boost (SIB) of 40 Gy in 10 fractions to 3 embedded high-dose spheres.

## Supplementary Tables

| Date | Neutrophil count (×10⁹/L) | Lymphocyte count (×10⁹/L) | NLR |
| --- | --- | --- | --- |
| 2025/4/17 | 10.87 | 0.50 | 21.74 |
| 2025/4/29 | 6.29 | 0.31 | 20.29 |
| 2025/5/2 | 6.08 | 0.16 | 38.00 |
| 2025/5/9 | 2.64 | 0.74 | 3.57 |
| 2025/5/29 | 4.49 | 2.15 | 2.09 |
| 2025/6/26 | 4.36 | 2.18 | 2.00 |
| 2025/8/31 | 3.87 | 3.37 | 1.15 |

**Supplementary Table 1.** Serial changes in neutrophil count, lymphocyte count, and NLR.
